# TXNDC12 inhibits pancreatic tumor cells ferroptosis by regulating GSH/GGT7 and promotes its growth and metastasis

**DOI:** 10.7150/jca.93208

**Published:** 2024-05-28

**Authors:** Xiangrong Xu, Yu Hei, Bobo Wang, Shuyue Tian, Xuanyu Chen, Jing Zhang, Fenghui Wang

**Affiliations:** 1 Yan'an University College of Basic Medical Sciences, Yan'an, 716000, China.; 2 Yan 'an City fungi resources Development and biological control key laboratory, Yan'an 716000, China.

**Keywords:** pancreatic cancer, TXNDC12, GGT7, GSH, ferroptosis, progression

## Abstract

**Background:** Thioredoxin domain-containing protein 12 (TXNDC12) is upregulated in a variety of tumours, including pancreatic cancer (PAAD), and its high expression is closely associated with poor prognosis. However, the regulatory mechanism of TXNDC12 in PAAD has not been reported. The aim of this study is to reveal the precise mechanism of TXNDC12 in regulating PAAD progression.

**Methods:** The expression of TXNDC12 in pan-cancer as well as PAAD was verified by TCGA and GTEx databases, Western blot and RT-qPCR. CCK8 assay, clone formation assay and cell cycle assay were used to observe the effect of TXNDC12 on the proliferation of PAAD cells, the migration and invasion capacities were verified by wound healing assay and Transwell assay. The effect of TXNDC12 on apoptosis of MIA PaCa-2 and PANC-1 cells was detected using Hochest and flow cytometry. Finally, the interaction of TXNDC12 with GGT7 was predicted by STRING database and confirmed by CO-IP assay, the effect of TXNDC12 on ferroptosis through GGT7 was evaluated by GSH assay, MDA assay, ROS assay and Western blot.

**Results:** TXNDC12 is upregulated in PAAD tissues, and patients with high TXNDC12 levels generally have shorter survival times. Knockdown of TXNDC12 significantly inhibited the proliferation, migration and invasion and promoted apoptosis of MIA PaCa-2 and PANC-1 cells. Mechanistically, knockdown of TXNDC12 resulted in a decrease in intracellular GSH content and an increase in GSSG content, as well as elevated levels of pro-ferroptosis factors, such as MDA and ROS. STRING database predicted that TXNDC12 interacts with GGT7, and CO-IP assay was used to validate this result. Finally, the effect of knocking down TXNDC12 on pancreatic cancer cell functions was able to be reversed by overexpression of GGT7.

**Conclusion:** TXNDC12 inhibits ferroptosis in PAAD cells through the GSH/GGT7 axis thereby promoting their development.

## Introduction

As a highly malignant tumour in the digestive system, PAAD has become one of the major public health problems worldwide due to its rapid progression and poor clinical prognosis 1. Currently, PAAD is mainly treated by surgery and radiotherapy, but problems such as chemotherapeutic drug resistance have led to a generally poor prognosis for patients, and some studies have confirmed that the development of chemotherapeutic drug resistance is closely related to apoptosis 2, so inducing non-apoptotic cells to die may provide an alternative therapeutic strategy for resisting apoptotic drug resistance.

Ferroptosis is a novel iron-dependent mode of cell death distinct from apoptosis and necrosis and is characterized by lipid peroxidation 3, a process that generates large amounts of Fe^2+^, lipid hydroperoxides (LOOH), and ROS, and is accompanied by a decrease in the activity of glutathione peroxidase 4 (GPX4) or glutathione (GSH) depletion. Some studies have confirmed that promoting ferroptosis shows great potential in tumour therapy, especially in eradicating aggressive malignancies that do not respond well to conventional therapies 4-6. For example, HLF can inhibit ferroptosis in Triple-negative breast cancer (TNBC) through the GGT1/GPX4 axis, thereby promoting cisplatin resistance in TNBC. GSH is an important component of the cellular antioxidant system and plays an important role in the treatment of tumours, and GSH can also trigger ferroptosis by interfering with cystine/glutamate reverse transporter proteins (systems Xc^-^), inactivating GPX4, or depletion of GSH 7. New studies have found that GSH depletion ferroptosis activating the pathway can effectively kill tumour cells 8, 9, and a nanopreparation based on arginine-rich manganese silicate nanobubbles has been invented, which is highly efficient at GSH depletion and can inhibit tumour development by inhibiting the activity of GPX4 10.

Thioredoxin domain-containing protein 12 (TXNDC12) is a thioredoxin structural domain-containing protein, also known as ERp16, ERp18, ERp19, or hTLP19, that is a member of the Protein Disulfide Isomerase (PDI) family and a member of the Thioredoxin (Trx) superfamily. The protein is widely expressed in all tissues, with the most abundant expression in liver and placenta 11. TXNDC12 maintains sulfhydryl homeostasis and prevents oxidative stress by encoding a protein involved in catalyzing the formation of disulfide bonds to reduce various intracellular disulfides 12. TXNDC12 can also be involved in endoplasmic reticulum stress by regulating the activation of ATF6α during the unfolded protein response by becoming a mixed disulfide with activating activated transcription factor 6α (ATF6α) 13, 14. In addition, several studies have confirmed that TXNDC12 also played an important role in cancer development and progression 15, 16, such as TXNDC12 promoted cell growth, migration, and invasion promoting tumorigenicity in human gastric cancer via FAK/ERK signaling pathway 17, TXNDC12 also promoted nuclear translocation of β-catenin promoting epithelial-mesenchymal transition process to promote metastasis and invasion of hepatocellular carcinoma 18. In glioma studies, TXNDC12 was found to be significantly overexpressed in gliomas, and this high expression was associated with the local immune microenvironment of gliomas, which predicted a poor prognosis for glioma patients 19. Although high expression of TXNDC12 can play a role in tumors, a study revealed that low mRNA levels of *TXNDC12* can also play a suggestive role in the poor prognosis of lung adenocarcinoma patients 20.

Currently, the relevant research of TXNDC12 in PAAD is still in the blank stage, so in this study we found that TXNDC12 promotes ferroptosis of PAAD cells by interacting with GGT7 and causing changes in GSH through bioinformatics analysis, cellular experiments and so on. Our findings may provide basic theoretical basis and experimental support for the discovery of effective targets or favorable factors for PAAD in clinical treatment and prognosis.

## Materials & Methods

### TXNDC12 expression in pan-cancer and Pancreatic cancer

The expression levels of *TXNDC12* mRNA in pan-cancer were analyzed using data obtained from TCGA (https://portal.gdc.cancer.gov/) and GTEx (https://www.gtexportal.org). Based on the above pan-cancer analysis, we downloaded pancreatic cancer-related data from the TCGA and GTEx databases and analysed *TXNDC12* expression levels in pancreatic cancer after removing duplicate and clinically insignificant samples. In addition, we further focused on the impact of *TXNDC12* expression on K-M curves in a database cohort of patients with PAAD, and data on PAAD cases was downloaded from GEPIA (http://gepia.cancer-pku.cn/) website.

### TXNDC12 KEGG enrichment assay

KEGG signalling pathway enrichment of TXNDC12 in PAAD was analysed via the LinkedOmics database (http://www.linkedomics.org). The false discovery rate (FDR) and the normalised enrichment score (NES) were used to rank the TXNDC12-related signalling pathways, and FDR≤0.05 was regarded as significantly enriched.

### TXNDC12 and GGT7 correlation

The proteins interacting with TXNDC12 were predicted using the STRING database (https://cn.string-db.org/) website, and based on this, we screened the pancreatic cancer-related data information in the TCGA database, and used Spearman correlation analysis to predict the expression of TXNDC12 and GGT7 in 178 cases of pancreatic cancer correlation, and the final results were shown as scatter plots.

### Cell culture and treatment

The human pancreatic tumor cell line PANC-1 was purchased from (Wuhan Doctoral Bioengineering Co., Ltd, Wuhan, China), MIA PaCa-2 was purchased from (iCell Bioscience Inc, Shanghai, China), ASPC-1 and human normal pancreatic ductal epithelial cells HPNE were preserved in the Medical Research and Experimental Centre of Yan'an University (Yan'an, China). PANC-1, MIA PaCa-2 and HPNE cells were cultured in DMEM (Biological Industries, Beit Haemek, Israel) containing 10% fetal bovine serum (FBS, Biological Industries, Beit Haemek, Israel). ASPC-1 cells were cultured in RPMI-1640 medium (Biological Industries, Beit Haemek, Israel) containing 10% FBS. All cells were cultured in a 5% CO_2_ incubator at 37 °C.

### Cell treatment

The siRNA fragment of human TXNDC12, negative control (NC), were chemically synthesized by GenePharma (Shanghai, China). The sequences were as follows:

si-1 (sense) 5'-GCAAAGCUCUAAAGCCCAATT-3', (antisense) 5'-UUGGGCUUUAGAGCUUUGCTT-3'; si-2 (sense) 5'-GGAUGAAGAGGAACCCAAATT-3', (antisense)5'-UUUGGGUUCCUCUUCAUCCTT-3'); NC (sense) 5'-UUCUCEGAACGUGUCACGUTT-3', (antisense) 5'-ACGUGACACGUUCGGAGAATT-3').

The negative vector (pcDNA3.1+), GGT7 overexpression vectors, TXNDC12 overexpression vectors, truncated TXNDC12 plasmid and the TXNDC12 mutant construct (both Cys66 and Cys69 were replaced by serine, CS) were also chemically synthesized by GenePharma (Shanghai, China). Transfection of overexpression vectors and small interfering RNA (siRNA) was performed by using the jet PRIME reagent (Polyplus-transfection SA), according to the manufacturer's protocol. For the effect of GSH on PAAD growth, cells were incubated with BSO (Sigma, USA) for 12 hours or GSH (Sigma, USA) for 3 hours before transfection.

### Western blot

Cells were lysed in lysis solution (Pioneer, Shanghai, China) supplemented with protease inhibitors and phosphatase inhibitors (Target Mol, USA). The protein concentration was determined using the BCA Protein Assay Kit (Pioneer, Shanghai, China), and then the protein samples were transferred onto PVDF membranes by SDS-PAGE electrophoresis and then incubated with specific primary antibodies at 4 °C overnight. Incubate with the corresponding anti-rabbit/anti-mouse secondary antibodies (Trans Gen Biotech, Beijing, China) for 1.5 h at room temperature. Luminescence signals were detected and recorded by Syngene GBox (Syngene, Cambridge, UK) using ECL (Boster Biological Technology co. ltd, Wuhan, China) in dark room. The primary and secondary antibodies used are listed as follows: GGT7 (abcam, ab273046, 1:1000), TXNDC12 (abcam, ab134938, 1:2000), CDK4 (Proteintech, 11,026-1-AP, 1:1000), CyclinD1 (Proteintech, 26,939-1-AP, 1:1000), CDK1 (Proteintech, 19532-1-AP, 1:1000), CyclinB1 (Proteintech, 55004-1-AP, 1:2000), Bax (Proteintech, 50599-2-Ig, 1:5000), Bcl-2 (Proteintech, 68103-1-Ig, 1:1000), GPX4 (AntiProtech, PA101995, 1:1000), 6*His (Proteintech, 66005-1-Ig, 1:10000), β-actin (Trans Gen Biotech, HC201-01, 1:4000), Goat Anti-Rabbit IgG (H+L) (Trans Gen Biotech, HS101-01, 1:4000), Goat Anti-Mouse IgG (H+L) (Trans Gen Biotech, HS201-01, 1:4000).

### Quantitative reverse transcription PCR(RT-qPCR)

Total RNA was extracted from the cells of each treatment group by the TRIzol method, and the cDNA was synthesized by reverse transcription using total RNA as a template with Reverse Transcription Kit (Takara Bio Inc, Japan) at 37°C for 15 min, 85 °C for 5 s, and 4 °C for 10 min. Quantitative real-time PCR was performed using 2×TransStart top Green qPCR Supermix (TransGen Biotech, Beijing, China) after pre-denaturation at 94°C for 5 min, 94 °C for 30 s, 58°C for 15 s, 72 °C for 30 s, and cycling for 40 times to obtain the cyclic values in each well. The measurements were analyzed by the relative quantification method 2^-ΔΔCt^ using *GAPDH* as the internal reference gene. The primer sequences were as follows: *TXNDC12*, 5'-GTCCTGCTGATTGTGAAAATGGC-3' (forward), 5'-TGATCCATGTCGAGGGTCAAA-3' (reverse); *GGT7*,5'-GCCTTGTGTTTGGGTATCGT-3' (forward), 5'-TGATCCATGTCGAGGGTCAAA-3' (reverse); *GAPDH*, 5'-GGAGCGAGATCCCTCCAAAAT-3' (forward), 5'-GGCTGTTGCATACTTCTCATGG-3' (reverse).

### CCK8 assay

The CCK8 assay was used to detect the proliferative capacity of the cells, MIA PaCa-2 and PANC-1 were spread in 96-well plates at 1500 cells/well, and then transfected after incubation for 24 hours (at this time it was 0 h), About 10 μL of CCK8 (TargetMol, USA) solution was added to the 96-well at 24, 48 and 72 h after transfection and cells were incubated in 37 °C for 2 h. The absorbance was measured on a microplate reader (MD, USA) at 450 nm.

### Clone formation assay

The pretreated cells were inoculated into 12-well plates at a density of 1000 cells/well. After 10-15 days of culture, the cells were fixed with 4% paraformaldehyde for 30 min after washing the medium with phosphate buffered saline (PBS), then stained with 0.1% crystal violet for 30 min, and the dye was washed and dried with PBS buffer, and the images were acquired after drying.

### Flow cytometry assay

MIA PaCa-2 and PANC-1 cells were inoculated in 6-well plates at 300,000/well and the cells were synchronized for 12 h after cell apposition. The cells were collected 24 h after transfection, digested with EDTA-free trypsin, washed twice with pre-cooled PBS and placed in 70% ethanol at -20 °C overnight. Then washed with pre-cooled PBS, resuspended for 15 min with 150 μL 0.2 mg/mL of RNaseA (Solarbio Science & Technology Co., Ltd. Beijing, China) and added 0.01 mg/mL propidium iodide (PI) dye (Solarbio Science & Technology Co., Ltd. Beijing, China) after RNaseA. The DNA content was detected by flow cytometry (Syngene, USA). Apoptosis was detected by Bestbio (Shanghai, China) apoptosis detection kit.

### Wound healing assay

Logarithmic growth phase cells were inoculated in 6-well plates at a density of 350,000/well and transfected after 24 h. Cells in each treatment group were scratched along the middle of the wells after 4-6 h of transfection, and the dislodged cells were rinsed with PBS, finally, photos were taken at 0 h, 24 h, 48 h, and 72 h. Pictures that were scratched in a straighter way, with a clean background, and with a consistent width and the same position at 0 h were selected for comparison, and the scratched pictures were statistically analyzed using the Image J software (National Institutes of Health, USA).

### Transwell assay

Transwell chambers were assayed for cell invasion ability by applying 100 μL of DMEM-diluted Matrigel gel (3:7 dilution) to the chambers to be checked and incubated at 37 °C for 30 min to form a gel, then pre-treated MIA PaCa-2 and PANC-1 cells were resuspended with DMEM and spread in small chambers at 50000/well, and the lower chamber was placed in 20% FBS DMEM, incubated at 37°C 5% CO_2_ for 48 hours. Cells were collected after 48 h of incubation, fixed with 4% paraformaldehyde for 30 min and stained with 0.1% crystal violet for 30 min before images were collected with an inverted microscope (Nikon, Tokyo, Japan). Migration experiments cells were collected after 24 h of incubation. The remaining experimental steps are the same as invasion, except for the Matrigel gel.

### GSH/GSSG, MDA, ROS assays

The pretreated cells were digested with EDTA-free trypsin and washed twice with PBS, the remaining operations were carried out according to the instructions of the S0053, S0033S and S0131S (Beyotime Biotechnology, Shanghai, China) kits.

### CO-IP

MIA PaCa-2 and HEK293 cells were spread at 2×10^6^ in 10 cm dishes and transfected 24 h later. Cells were lysed using Mammalian cell lysis reagent (Pioneer, Shanghai, China) at 48 h post-transfection, and the extracted proteins were divided into 3 groups including Input, IgG and IP groups, to which 20 μL of Protein A+G magnetic beads (Beyotime, Shanghai, China) and 5 μg of antibody (IgG or the corresponding primary antibody) were added respectively and bound on a vertical rotator at 4 °C for 12-18 h. Then the supernatant was discarded, the beads were washed three times with 1×TBS, and 50 μL of buffer was added to the beads at 100 °C for 10 min. Finally, the processed protein samples were detected by Western blot.

### Statistical analysis

The experimental data were statistically analyzed using SPSS 26.0 software. All experiments were performed with three independent replicates. A two-group comparison was determined by Student's t test and the one-way analysis of variance (ANOVA) followed was performed for multigroup comparison, *P* < 0.05 was considered to be statistically significant.

## Results

### TXNDC12 is upregulated in pancreatic cancer tissues and cells

**Figure 1:**
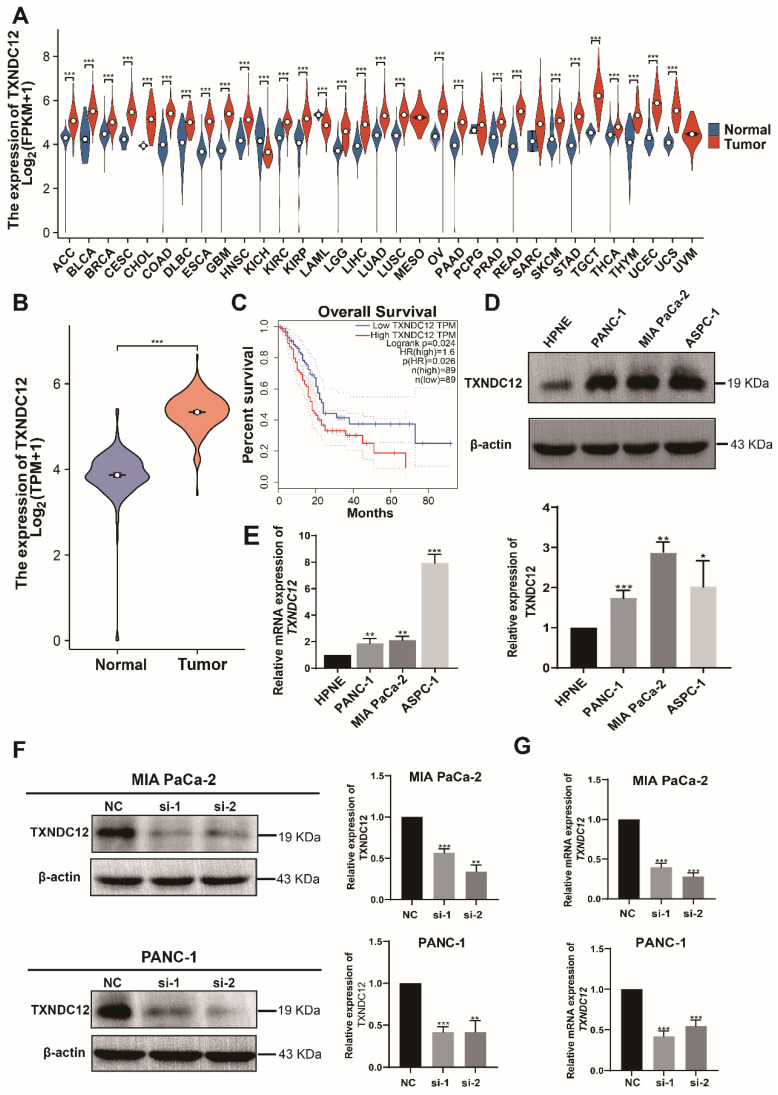
TXNDC12 is upregulated in pancreatic tumor tissues and cells. (A). mRNA expression of *TXNDC12* in pan-cancer in TCGA and GTEx database, (B). mRNA expression of *TXNDC12* in pancreatic tumor jointly analyzed by TCGA and GTEx (T=183, N=167), (C). K-M curves of PAAD patients based on *TXNDC12* expression levels, (D). TXNDC12 expression level of TXNDC12 in normal pancreatic cells and PAAD cells, (E). *TXNDC12* mRNA expression level in normal pancreatic cells and PAAD cells, (F). Knockdown of TXNDC12 protein expression level in MIA PaCa-2, PANC-1, (G). Knockdown of *TXNDC12* mRNA expression level in MIA PaCa-2, PANC-1, ^*^P<0.05, ^**^P<0.01, ^***^P<0.001.

Previous studies have shown that TXNDC12 is highly expressed in a variety of tumors 19-21, so we first analysed the expression of *TXNDC12* in pan-cancer and the results showed that *TXNDC12* expression levels were up-regulated in tissues of most cancer types, including PAAD, compared to normal tissues (Fig. 1A). Then we screened pancreatic cancer-related information through TCGA and GTEx databases and analysed the expression of *TXNDC12* in pancreatic cancer, and the results showed that *TXNDC12* expression was upregulated in pancreatic cancer tissues(Fig. 1B). As shown in the K-M curves, PAAD patients with high expression of *TXNDC12* had a lower overall survival than those with low *TXNDC12* expression (Fig. 1C). Moreover, Western blot and RT-qPCR assay showed that the protein and mRNA expression levels of TXNDC12 were significantly higher in PAAD cells than that in normal pancreatic epithelial cells (Fig. 1D, E).

### TXNDC12 promotes the proliferation of PAAD cells

**Figure 2:**
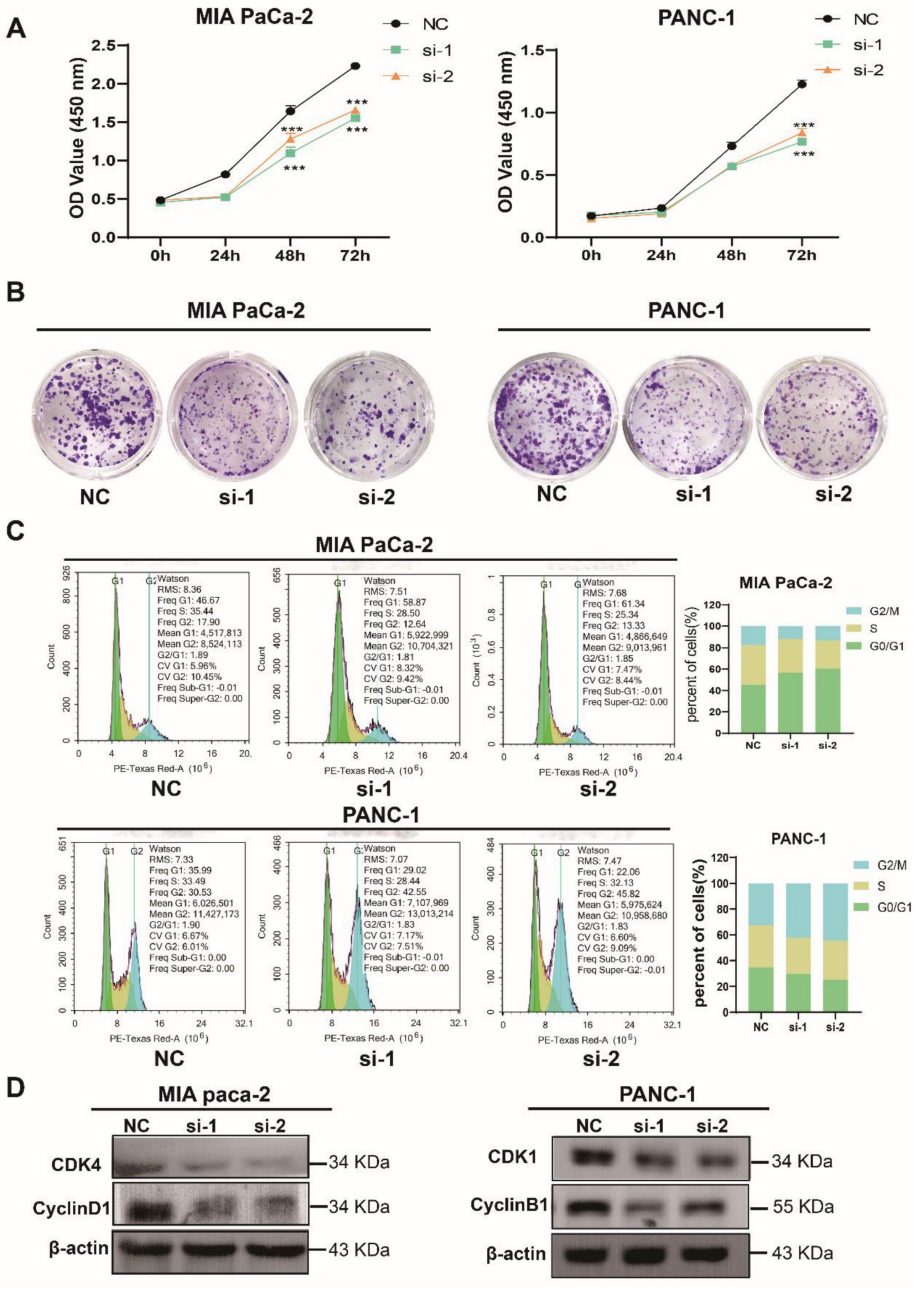
Effect of knockdown of TXNDC12 on proliferation of PAAD cells. (A). Cell viability assay of MIA PaCa-2 and PANC-1 cells after knockdown of TXNDC12, (B). Effect of knockdown of TXNDC12 on the clone forming ability of MIA PaCa-2 and PANC-1cells, (C). Effect of knockdown of TXNDC12 on the cell cycle of MIA PaCa-2 and PANC-1, (D). Western blot detection of the effect of knockdown of TXNDC12 on the cell cycle-related proteins of MIA PaCa-2 and PANC-1, ^***^P<0.001.

To further elucidate the role of TXNDC12 in PAAD, we knocked down TXNDC12 in MIA PaCa-2 and PANC-1 cell lines and verified the knockdown efficiency by Western blot and RT-qPCR (Fig. 1F, G). As shown in (Fig. 2A), the proliferative ability of MIA PaCa-2 and PANC-1 cells was gradually weakened after knocked down TXNDC12. Clone formation assay showed that number of clones formed by MIA PaCa-2 and PANC-1 cells was significantly reduced after knockdown of TXNDC12 (Fig. 2B). The results of cell cycle assay showed that knockdown of TXNDC12 lead MIA PaCa-2 cell cycle block in G0/G1 phase, whereas, PANC-1 cells were blocked in G2/M phase (Fig. 2C, D).

### TXNDC12 promotes the migration and invasion of PAAD cells

**Figure 3:**
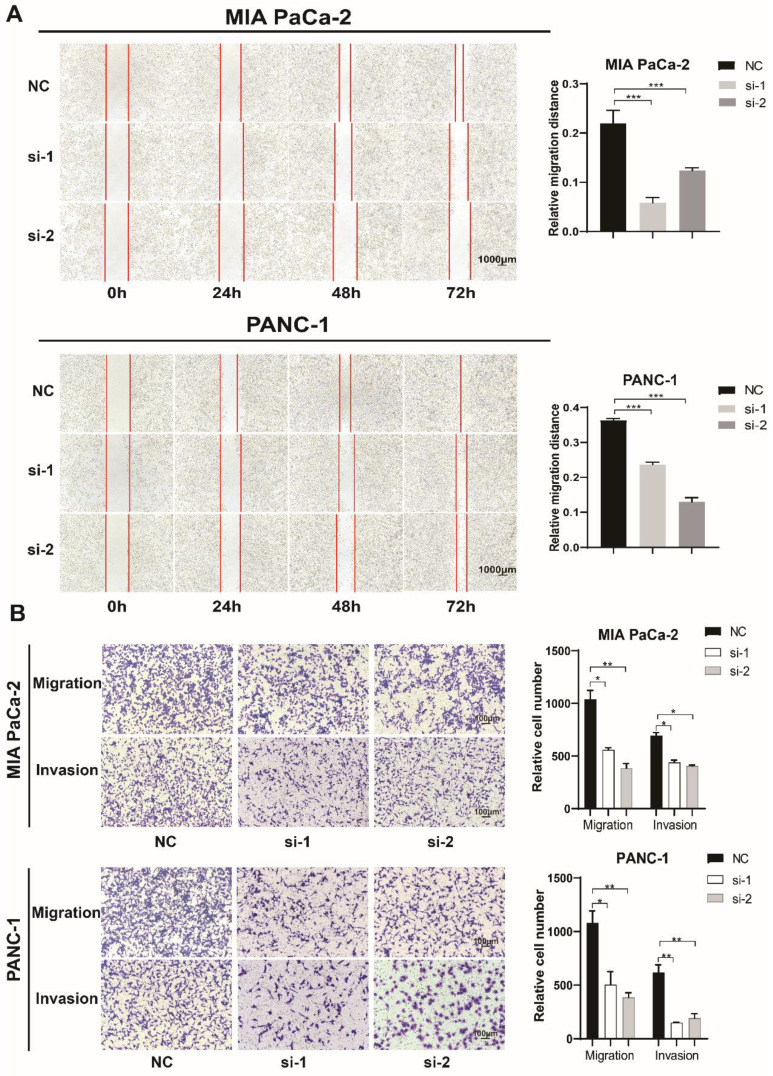
Effect of knockdown of TXNDC12 on PAAD cells migration. (A). Wound healing assay to detect MIA PaCa-2 and PANC-1 migratory ability after knockdown of TXNDC12 (Scale bar = 1000μm), (B). Transwell assay to detect MIA PaCa-2, PANC-1 migratory and invasive ability after knockdown of TXNDC12 (scale bar = 100μm), ^*^P<0.05, ^**^P<0.01, ^***^P<0.001.

In order to prove the effect of TXNDC12 on the migratory capacity of PAAD cells, we performed a wound healing experiment, and the results (Fig. 3A) showed that knockdown of TXNDC12 significantly inhibited the migration ability of MIA PaCa-2 and PANC-1 cells (*P*<0.01). The Transwell assay demonstrated that knockdown of TXNDC12 significantly inhibited the migration and invasion ability of MIA PaCa-2 and PANC-1cells (Fig. 3B).

### Knockdown of TXNDC12 promotes apoptosis in PAAD cells

**Figure 4:**
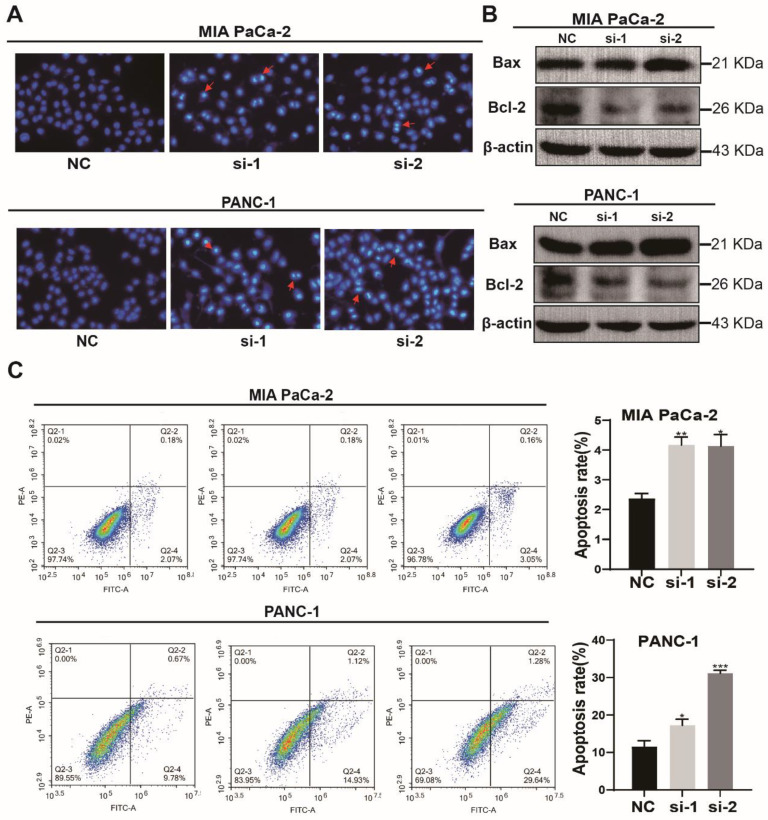
Effect of knockdown of TXNDC12 on apoptotic capacity of PAAD cells. (A). The image of the Hochest apoptosis assay (×10), (B). Western blot for apoptosis-related protein expression after knockdown of TXNDC12, (C). flow cytometry for apoptosis levels of MIA PaCa-2 and PANC-1 after knockdown of TXNDC12, ^*^P<0.05, ^**^P<0.01, ^***^P<0.001.

Then, the effect of knockdown of TXNDC12 on the apoptotic capacity of PAAD cells was detected using Hochest staining. As shown in (Fig. 4A), the number of MIA PaCa-2 and PANC-1 apoptotic cells was increased after knockdown of TXNDC12. Flow cytometry results showed an increased percentage of apoptotic cells after knockdown of TXNDC12 (Fig. 4C). To further verify that PAAD cell apoptosis was affected by TXNDC12, we examined apoptosis-related proteins by Western blot. The results suggested that the expression of apoptosis-promoting proteins was significantly increased after knocking down TXNDC12. On the contrary, the expression of apoptosis-suppressing proteins was decreased (Fig. 4B).

### TXNDC12 regulates ferroptosis through glutathione metabolism

**Figure 5:**
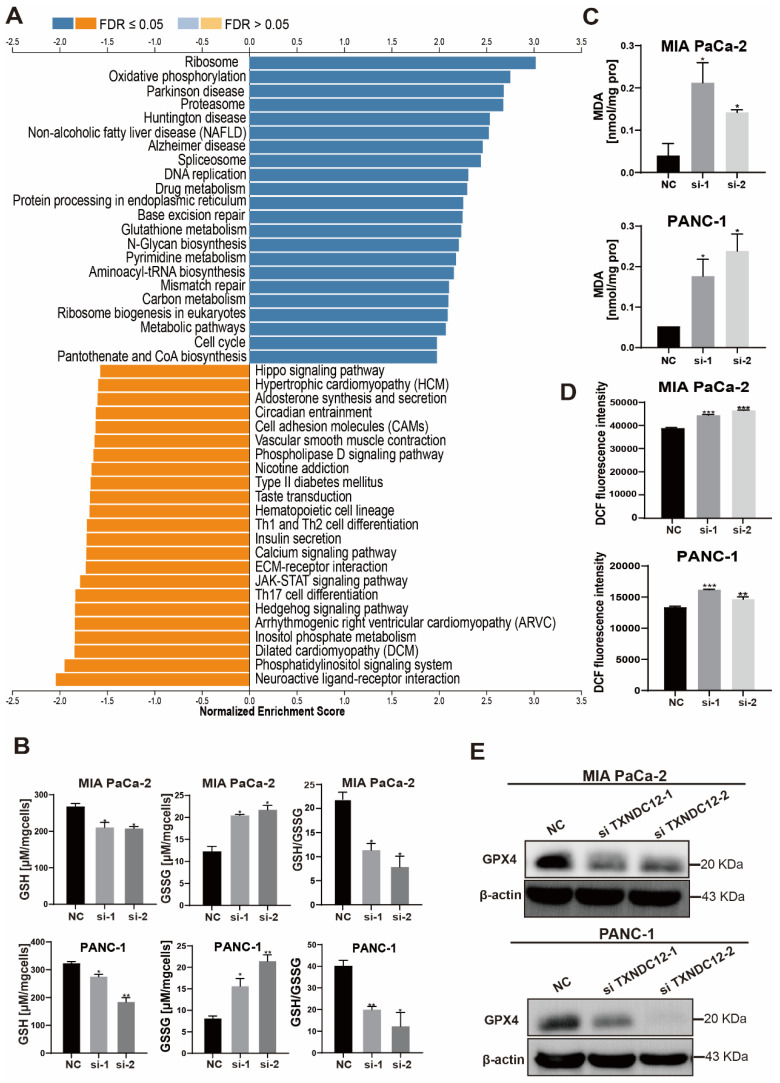
TXNDC12 regulates ferroptosis through glutathione metabolism. (A). KEGG enrichment analysis of the pathways through which *TXNDC12* may be involved in PAAD development, (B). changes in GSH and GSSG content after knockdown of TXNDC12, (C). MDA content changes after knocking down TXNDC12, (D). Changes in ROS after knockdown of TXNDC12, (E). Changes in GPX4 protein expression after knockdown of TXNDC12 by Western blot detection, ^*^P<0.05, ^**^P<0.01, ^***^P<0.001.

GSH, as an indispensable nutrient for cell growth, is important for tumour development, and GSH depletion not only plays an important role in cancer therapy but also promotes ferroptosis 22. We first performed KEGG analysis through the LinkedOmics website (Fig. 5A) and found that TXNDC12 is involved in regulating glutathione metabolism. Then, we detected the changes of intracellular GSH and GSSG contents after knocking down TXNDC12, and the results showed (Fig. 5B) that the intracellular GSH content was decreased while GSSG content increased after knocking down TXNDC12. Since GSH depletion has been shown to promote ferroptosis, we examined the effects of knocking down TXND12 on ferroptosis in PAAD cells, and the results showed that knocking down TXNDC12 increased the levels of ROS, MDA, and decreased the expression of GPX4 (Fig. 5C-E). To further confirmed that TXNDC12 inhibits PAAD cell ferroptosis via GSH metabolism, we used the GSH synthesis inhibitor BSO (PANC-1 1.53 mM, MIA PaCa-2 3.506 mM) and exogenous GSH 500 μM to incubate the cells and detected changes in the content of GSH and GSSG while knocking down TXNDC12. As shown in (Supplementary Fig. 1A), GSH content was increased and GSSG content decreased after GSH supplementation, but both GSH and GSSG contents were decreased after the addition of BSO. In addition, ROS, MDA and Western blot assays showed that GSH partially reduced the promotion of MDA, ROS and the inhibitory effect of GPX4 by knockdown TXNDC12, while BSO had the opposite effect (Supplementary Fig. 1B-E). The above experimental results show that TXNDC12 inhibits ferroptosis in PAAD cells by affecting glutathione metabolism.

### Mutations in TXNDC12 active site cysteine block GGT7 activation to inhibit ferroptosis in PAAD cells

**Figure 6:**
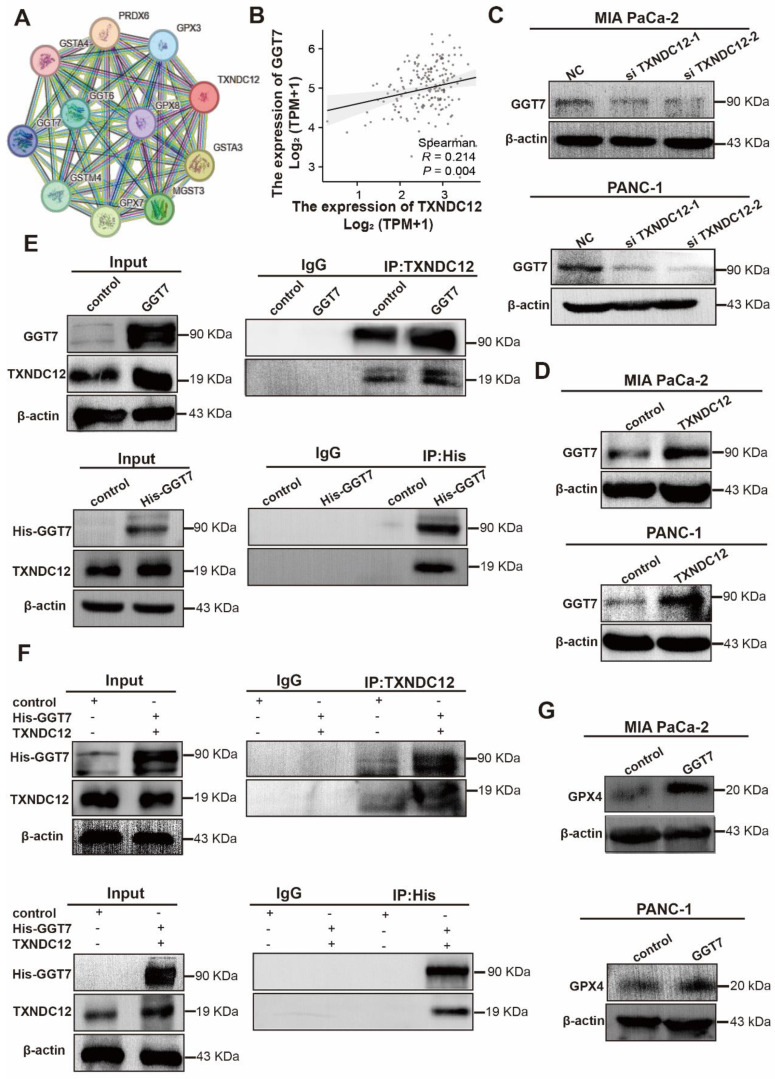
TXNDC12 regulates ferroptosis in PAAD cells through GGT7. (A). STRING database predicts TXNDC12 interacting proteins, (B). TCGA database predicts TXNDC12 correlation with GGT7, (C) and (D). Western blot detection of GGT7 expression changes after knockdown or overexpression of TXNDC12, (E). Overexpression of GGT7 in MIA PaCa-2 and fishing for GGT7 or TXNDC12 using TXNDC12 or His-tagged antibody as bait protein, (F). Overexpression of both GGT7 and TXNDC12 in HEK293 fishing for TXNDC12 or GGT7 using His-tagged or TXNDC12 antibody as bait protein, (G). Changes in GPX4 expression after overexpression of GGT7 detected by Western blot.

**Figure 7:**
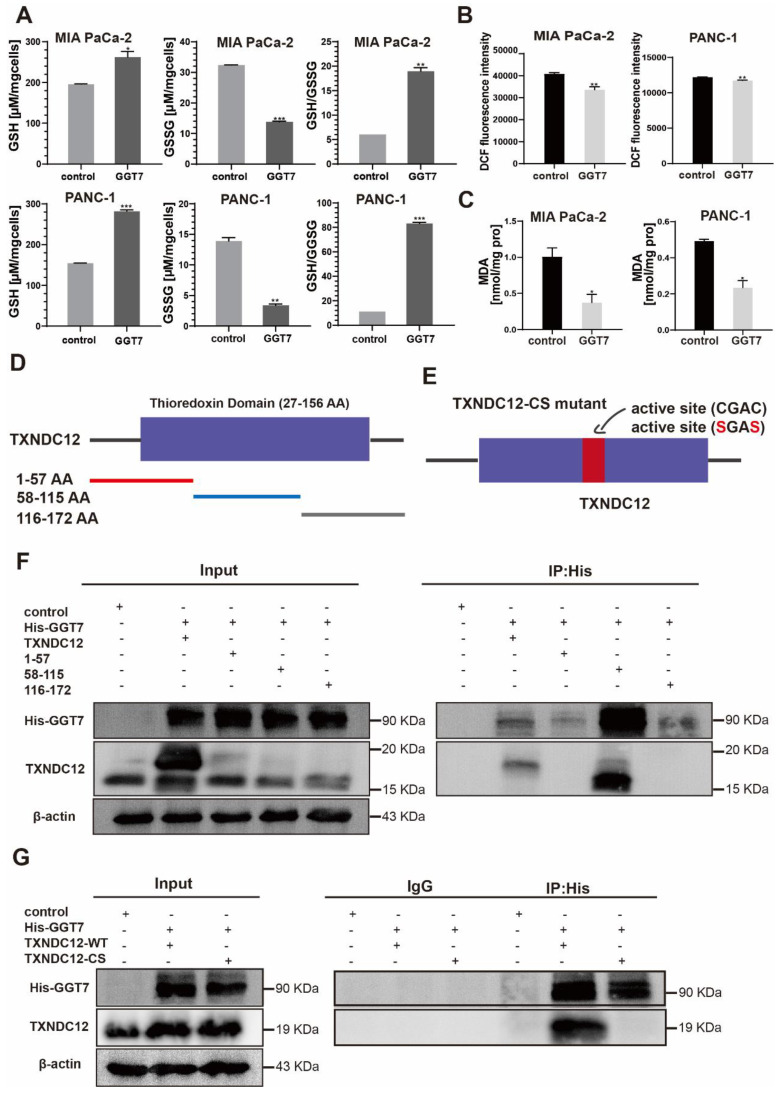
TXNDC12 interacts with GGT7 via the thioredoxin-like domain. (A). Changes in GSH, GSSG content in MIA PaCa-2 and PANC-1 cells detected after overexpression of GGT7, (B). Changes in ROS content in MIA PaCa-2 and PANC-1 cells after overexpression of GGT7, (C). Changes in MDA content in MIA PaCa-2 and PANC-1 cells after overexpression of GGT7, (D) and (E). TXNDC12 truncation and a map of mutation patterns, (F). HEK 293 cells transfected with GGT7 and full-length TXNDC12 or TXNDC12 fragments were immunoprecipitated with an anti-His antibody, (G). MIA PaCa-2 transfected with GGT7, wild-type TXNDC12 (WT), or cysteine-mutated TXNDC12 (CS) were immunoprecipitated with an anti-His antibody, ^*^P<0.05, ^**^P<0.01, ^***^P<0.001.

Although glutathione depletion has been shown to promote the occurrence of ferroptosis, the regulation of ferroptosis by TXNDC12 as an upstream regulator of glutathione metabolism is unknown. To explore the mechanism how TXNDC12 regulates ferroptosis in PAAD cells through GSH, we predicted the proteins that might interact with TXNDC12 by the STRING database and screened GGT7 from them (Fig. 6A). and then found that TXNDC12 was positively correlated with GGT7 by according to the TCGA database (Fig. 6B). Western blot results showed (Fig. 6C, D) that GGT7 expression was downregulated after knockdown of TXNDC12 and upregulated after overexpression of TXNDC12. CO-IP results showed (Fig. 6E, F) that TXNDC12 interacted with GGT7 both in MIA PaCa-2 and HEK293 cells, suggesting that TXNDC12 may activate GGT7 through protein-protein interaction. Subsequently, we examined the changes in the content of GSH, GSSG, ROS, and MDA, after overexpression of GGT7. The results showed that the content of GSH was increased while the contents of GSSG, ROS, and MDA were decreased by overexpression of GGT7, and GPX4 expression levels were significantly higher than control (Fig. 6G, Fig. 7A-C). To further explore the mechanism of action of TXNDC12 with GGT7, we mapped the binding domains of TXNDC12 by transfection of TXNDC12 truncation mutants into HEK293 cells. As shown in (Fig. 7D, F), only the fragments containing the active site of the thioredoxin domain (58-115) interacted with GGT7, indicating that the structural domain of TXNDC12 is essential for the interaction between TXNDC12 and GGT7. On this basis, to further investigate whether the PDI enzymatic activity of TXNDC12 is required for GGT7 activation, we constructed a TXNDC12-CS mutant in which both cysteines in the active site were mutated. As shown in (Fig. 7E, G), the interaction between TXNDC12 and GGT7 was eliminated by the mutation. Taken together, these results demonstrated that the promotion of GSH and the inhibition of ferroptosis by TXNDC12 are regulated by interaction with GGT7, and the TXNDC12 active site is crucial for the interaction of TXNDC12 with GGT7.

### TXNDC12 promotes PAAD cells development through suppressing GGT7-mediated ferroptosis

**Figure 8:**
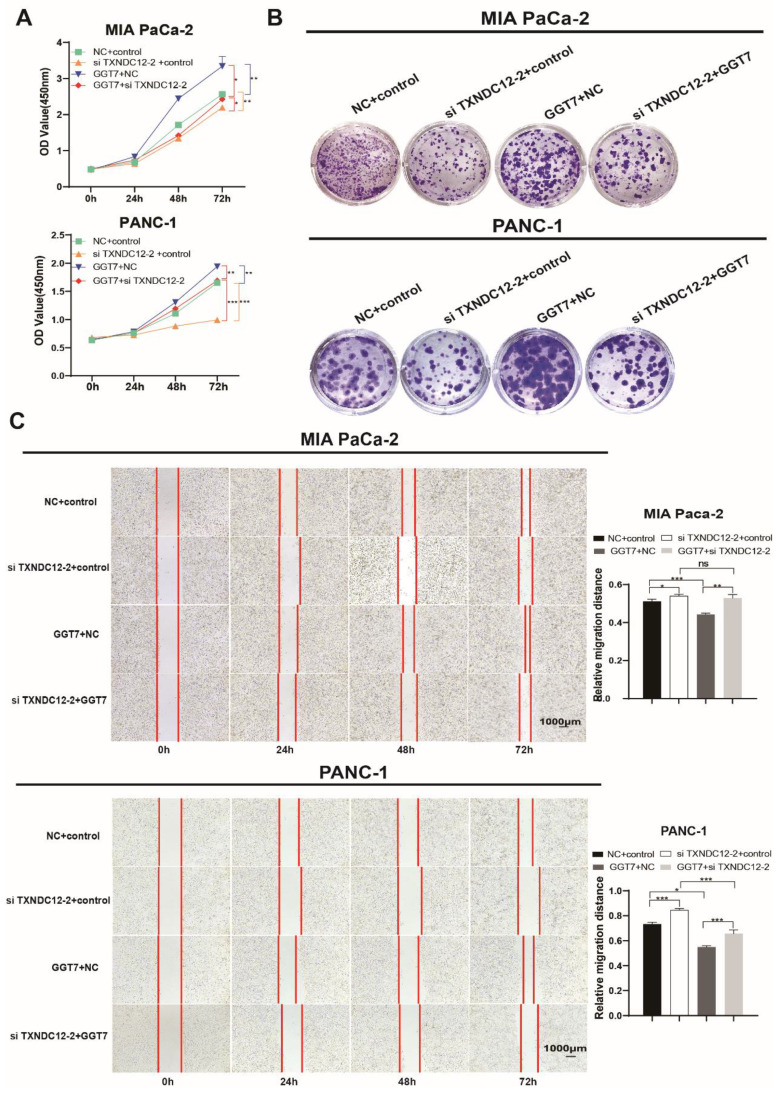
Effect of TXNDC12 and GGT7 together on the function of PAAD cells. (A). Effects of knockdown of TXNDC12 in combination with overexpression of GGT7 on the proliferation of MIA PaCa-2 and PANC-1 cells, (B). Effects of knockdown of TXNDC12 in combination with overexpression of GGT7 on the clone formation ability of MIA PaCa-2 and PANC-1, (C). Effects of knockdown of TXNDC12 in combination with overexpression of GGT7 on the migration level of MIA PaCa-2 and PANC -1 (scale bar = 1:1000μm), ^*^P<0.05, ^**^P<0.01, ^***^P<0.001, ns indicates that the difference is not statistically significant.

**Figure 9:**
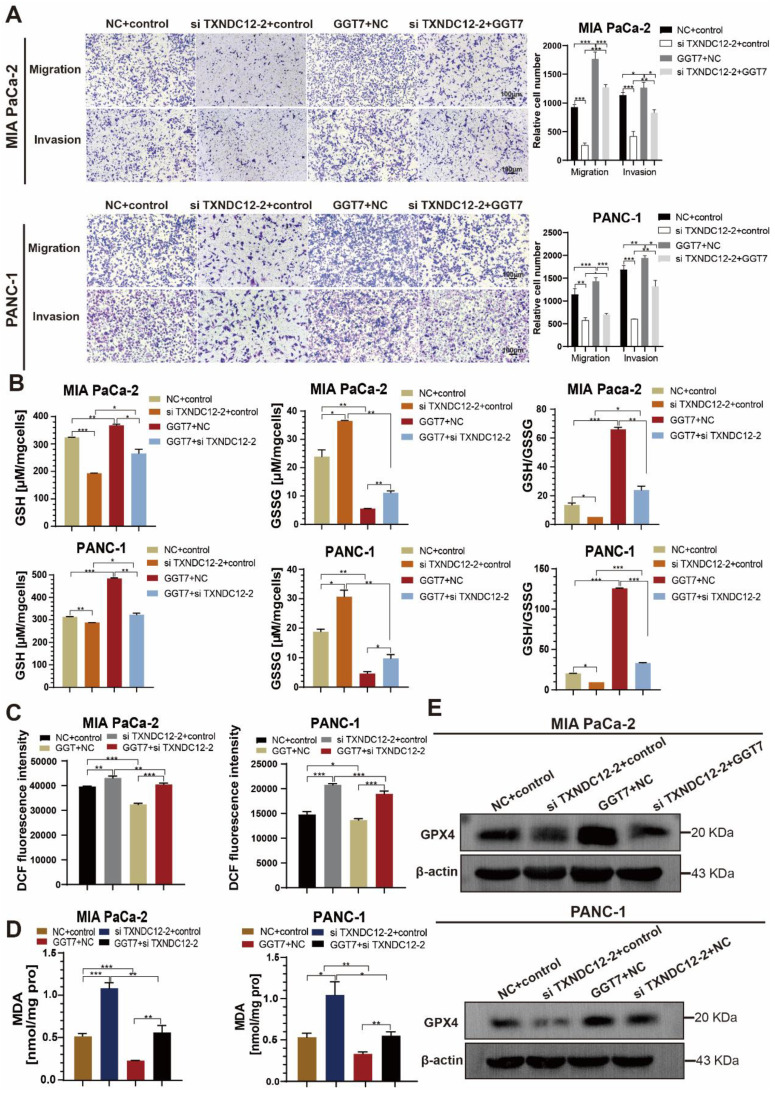
Effect of knockdown of TXNDC12 in combination with overexpression of GGT7 on GSH as well as ferroptosis. (A). Transwell assay to detect MIA PaCa-2 and PANC-1 migration as well as invasion ability (scale bar =1:100μm), (B). Knockdown of TXNDC12 while overexpressing GGT7 on GSH as well as GSSG, (C). Knockdown of TXNDC12 while overexpressing GGT7 to detect the intracellular ROS levels in MIA PaCa-2 and PANC-1 cells, (D). Measurement of intracellular MDA content after co-treatment of TXNDC12 and GGT7, (E). Western blot to detect the GPX4 expression levels in MIA PaCa-2 and PANC-1, ^*^P<0.05, ^**^P<0.01, ^***^P<0.001.

γ-glutamyl transpeptidase 7 (GGT7) is a newly discovered enzyme involved in glutathione metabolism, which mainly regulates the redox function in the human body and plays an important role in the synthesis and catabolism of glutathione 23. Wang X. showed that GGT7 could directly bind to RAB7 to induce autophagy, inhibited ROS and MAPK cascade responses, and played a key tumour-suppressive role in gastric cancer 24. On the contrary, our results showed that GGT7 is upregulated in MIA PaCa-2 and PANC-1 cells, and overexpression of GGT7 can promote the proliferation and migration of pancreatic cancer cells (Supplementary Fig. 2). This may be related to tumor heterogeneity. In addition, we also found that overexpression of GGT7 reversed the inhibitory effect of si TXNDC12-2 on the proliferation of PAAD cells using CCK8 assay, clone formation assay (Fig. 8A, B). The results of wound healing assay showed that overexpression of GGT7 could reverse the inhibitory effect of si TXNDC12-2 on the migration of PAAD cells (Fig. 8C), meanwhile, the Transwell results indicated that overexpression of GGT7 partially restored the inhibitory effect of knockdown of TXNDC12 on the migration as well as invasion of MIA PaCa-2 and PANC-1 cells (Fig. 9A).

Furthermore, in order to investigate the effects of overexpression of GGT7 in combination with knockdown of TXNDC12 on GSH content as well as ferroptosis in PAAD cells, the authors performed GSH, MDA, and ROS content measurements, and the results showed that overexpression of GGT7 could partially reverse the knockdown of the inhibitory effect of TXNDC12 on GSH and the promotional effect on MDA, ROS compared to si TXNDC12-2 group (Fig. 9B-D). Western blot results showed that overexpression of GGT7 could partially revert the inhibitory effect of si TXNDC12-2 on GPX4 expression (Fig. 9E). In a word, TXNDC12 inhibits PAAD cell ferroptosis by regulating GSH/GGT7 and promotes their growth and metastasis.

## Discussion

Pancreatic tumour still lacks effective treatment because of its high degree of malignancy and high susceptibility to metastasis. At present, the effective chemotherapy drugs for PAAD are still gemcitabine, capecitabine, paclitaxel, etc. Although the efficacy of these drugs is significantly, but the side effects and drug resistance problems have not been solved 25. Therefore, there is a need to develop novel therapeutic strategies specifically targeting PAAD to prolong patient survival time and improve prognosis.

Recent studies have demonstrated that PDI family members play important roles in cancer development and progression. Endoplasmic reticulum protein 5(ERp5) can enhance immune escape by blocking NKG2D ligand recognition in Hodgkin's lymphoma thereby promoting disease progression 26. Inhibition of recombinant endoplasmic reticulum resident protein 57 (ERp57) increases the protein defolding response and promotes the activation of PERK, which ultimately leads to increased levels of apoptosis in breast cancer cells 27. TXNDC12, as one of the PDI family members, is abnormally expressed in tumours such as gastric cancer and hepatocellular carcinoma, but the role of TXNDC12 in PAAD remains unknown. Our results show that TXNDC12 is upregulated in PAAD, and knockdown of TXNDC12 can inhibit the biological functions of PAAD cells such as proliferation, migration and invasion. We also found that TXNDC12 regulation of PAAD is mediated by inhibition of ferroptosis.

GSH has been shown to act as a reductive co-substrate for GPX4, inducing GPX4 inactivation and thereby triggering ferroptosis in tumour cells. In addition, GSH can also affect intracellular cysteine levels and GPX4 activity by inhibiting the Xc^-^ system 8. The targeted Xc^-^ system inactivates GPX4 or depletes GSH, thereby triggering ferroptosis 28. However, little is known about the effect of GSH on the biological function of PAAD. In this study, we found that it was not the higher concentration of GSH that promoted tumour cells more significantly, but higher concentrations of GSH had an inhibitory effect on tumour cells instead (Supplementary Fig. 3). This may be related to the production of NH_3_ by GSH catabolism and the negative feedback regulation of the organism, and the specific mechanism is still unclear, which is worthy of our further investigation. Different concentrations of GSH have opposite effects on tumor cells adding to the complexity of the possible use of GSH in clinical treatment, warranting further studies. Besides, our study found that TXNDC12 regulates PAAD ferroptosis via GSH, and since we proved that TXNDC12 inhibits PAAD ferroptosis, we hypothesised that TXNDC12 may influence ferroptosis via GSH. To rule out the possibility that GSH is dispensable for PAAD ferroptosis inhibited by TXNDC12, we utilized specific inhibitor of GSH synthesis and exogenous GSH, and concluded that GSH is required for the inhibition of PAAD ferroptosis by TXNDC12.

Gamma-glutamyl transpeptidase (GGT) hydrolyses glutamate in GSH or GSSG to produce Gly, Cys and Cys_2_ for cellular uptake of amino acids. Inhibition of GGT inhibits the activity of the Xc^-^ system and thus promotes ferroptosis. GGT1, a member of the GGT family, has been shown to inhibit ferroptosis via the GSH/GPX4 axis and thus inhibit tumour progression 6, 29, and its amino acid sequence shares 34% homology with its fellow family member GGT7, the protein space structure also has similarity. We speculate that GGT7 has a similar function to it and can be regulated by TXNDC12 thereby inhibiting PAAD ferroptosis. To verify this hypothesis, we confirmed the interaction between TXNDC12 and GGT7 by immunoprecipitation, and verified that the inhibitory effect of TXNDC12 on ferroptosis in PAAD cells was triggered by GGT7. Collectively, the results of our study linked TXNDC12 with GGT7, thus clarifying the underlying mechanism of TXNDC12-mediated PAAD growth and metastasis. However, the detailed mechanisms by which the interaction between TXNDC12 and GGT7 to regulate ferroptosis still requires further investigation.

## Conclusions

In conclusion, our study demonstrated a novel role for TXNDC12 in the regulation of ferroptosis through interaction with GGT7 and GSH metabolism, and the study of the biological function and potential molecular mechanisms of TXNDC12 in PAAD could provide a theoretical basis and experimental rationale for the development of new bioprosthetic markers or the development of targeted drugs in PAAD.

## Supplementary Material

Supplementary figures.
