# Supplementary material for: TXNDC12 inhibits pancreatic tumor cells ferroptosis by regulating GSH/GGT7 and promotes its growth and metastasis
Source: J Cancer. 2024 May 28;15(12):3913–29. doi: 10.7150/jca.93208 (PMC11190766; doi:10.7150/jca.93208)
Supplement: Supplementary file 1 — Supplementary figures. [file jcav15p3913s1.pdf]

Supplementary Fig.1 TXNDC12 inhibits in pancreatic cancer cells ferroptosis via GSH

(A). Changes in GSH and GSSG content after knockdown of TXNDC12 in combination with GSH, BSO, (B). Changes in MDA content after knockdown of TXNDC12 in combination with GSH, BSO, (C). Changes in ROS content after knockdown of TXNDC12 in combination with GSH, BSO, (D) and (E). GPX4 protein expression after knockdown of TXNDC12 combination with GSH or BSO, $^{*}P<0.05$, $^{**}P<0.01$, $^{***}P<0.001$, ns indicates that the difference is not statistically significant.

Supplementary Fig. 2 GGT7 expression and function in PAAD

(A). Protein expression level of GGT7 in normal pancreatic epithelial cells versus PAAD cells, (B). mRNA expression level of *GGT7* in normal pancreatic epithelial cells versus PAAD cells, (C) and (D). Overexpression efficiency of GGT7 in MIA PaCa-2 and PANC-1 cells detected by Western blot and RT-qPCR, (E). Clone formation assay to detect the effect of overexpression of GGT7 on the clone formation ability of selected PAAD cells, (F). Wound healing assay to detect the effect of overexpression of GGT7 on the migration level of PAAD cells, (G). CCK8 assay to detect the effect of GGT7 on the proliferative ability of MIA PaCa-2 and PANC-1 cells, $^{*}P<0.05$, $^{**}P<0.01$, $^{***}P<0.001$.

Supplementary Fig. 3 Effect of GSH on the proliferative capacity of PAAD cells

(A). CCK8 assay to detect the effect of different concentrations of GSH on the proliferation level of MIA PaCa-2 and PANC-1 cells at different times, (B). CCK8 assay to detect the effect of different concentrations of BSO on the proliferative capacity of pancreatic cancer cells, (C) and (D). Clone formation assays to detect the effects of different concentrations of GSH and BSO on the clone forming ability of MIA PaCa-2 and PANC-1 cells, (E). CCK8 assay to detect the effect of knockdown of TXNDC12 along with GSH supplementation on the proliferative capacity of PAAD cells, (F). CCK8 assay to detect changes in the proliferation level of MIA PaCa-2 and PANC-1 cells after knockdown of TXNDC12 following depletion of GSH in these cells by BSO, (G) and (H). Clone formation assays to detect changes in clone formation in MIA PaCa-2 and PANC -1 cells for changes in clone formation, $^{*}P<0.05$, $^{**}P<0.01$, $^{***}P<0.001$.

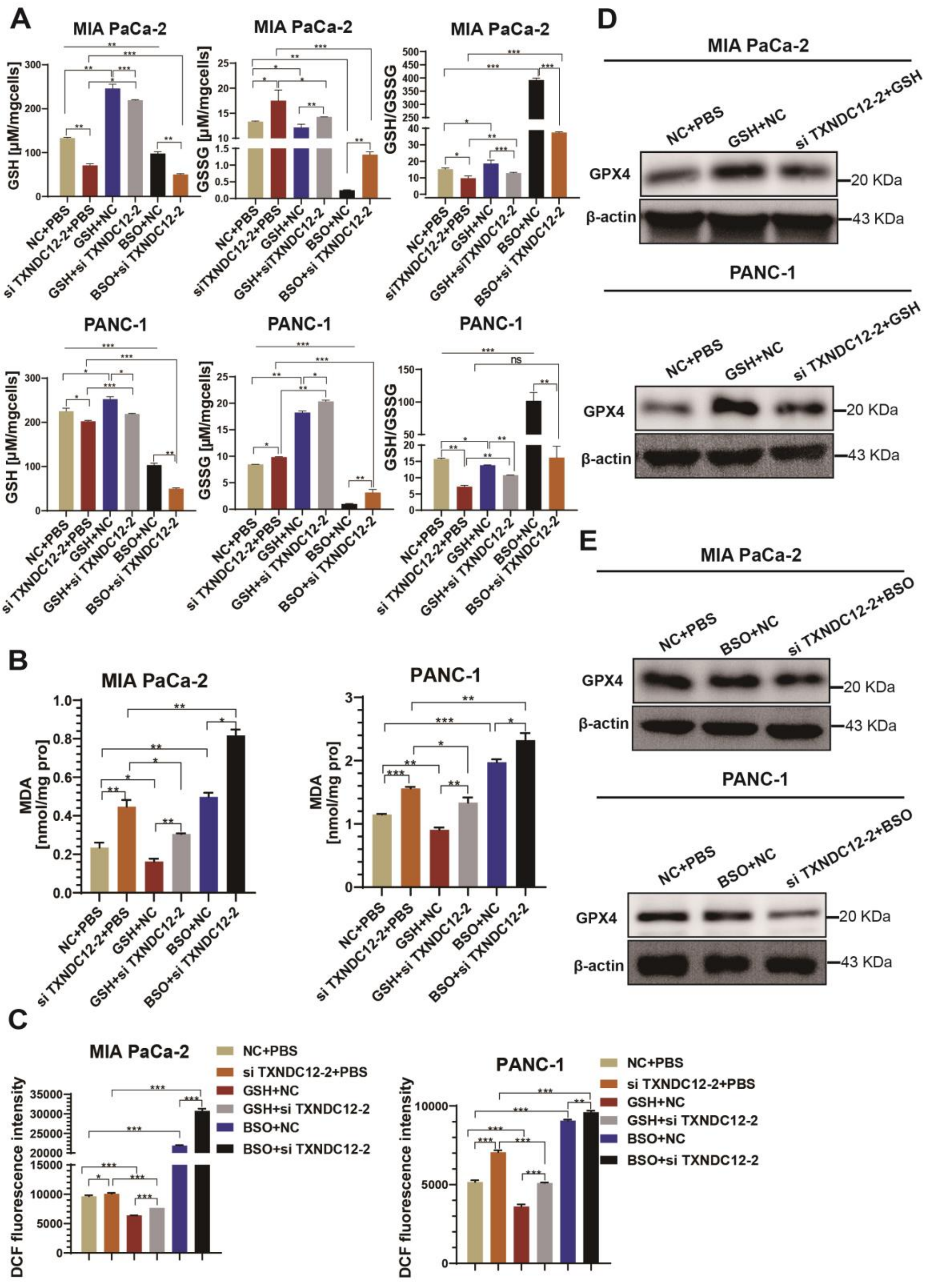
A
MIA PaCa-2
GSH [μM/mgcells]
GSSG [μM/mgcells]
GSH/GSSG
PANC-1
NC+PBS
si TXNDC12-2+PBS
GSH+NC
GSH+si TXNDC12-2
BSO+NC
BSO+si TXNDC12-2
B
MDA [nmol/mg pro]
C
DCF fluorescence intensity
D
GPX4
β-actin
20 KDa
43 KDa
si TXNDC12-2+GSH
E
si TXNDC12-2+BSO

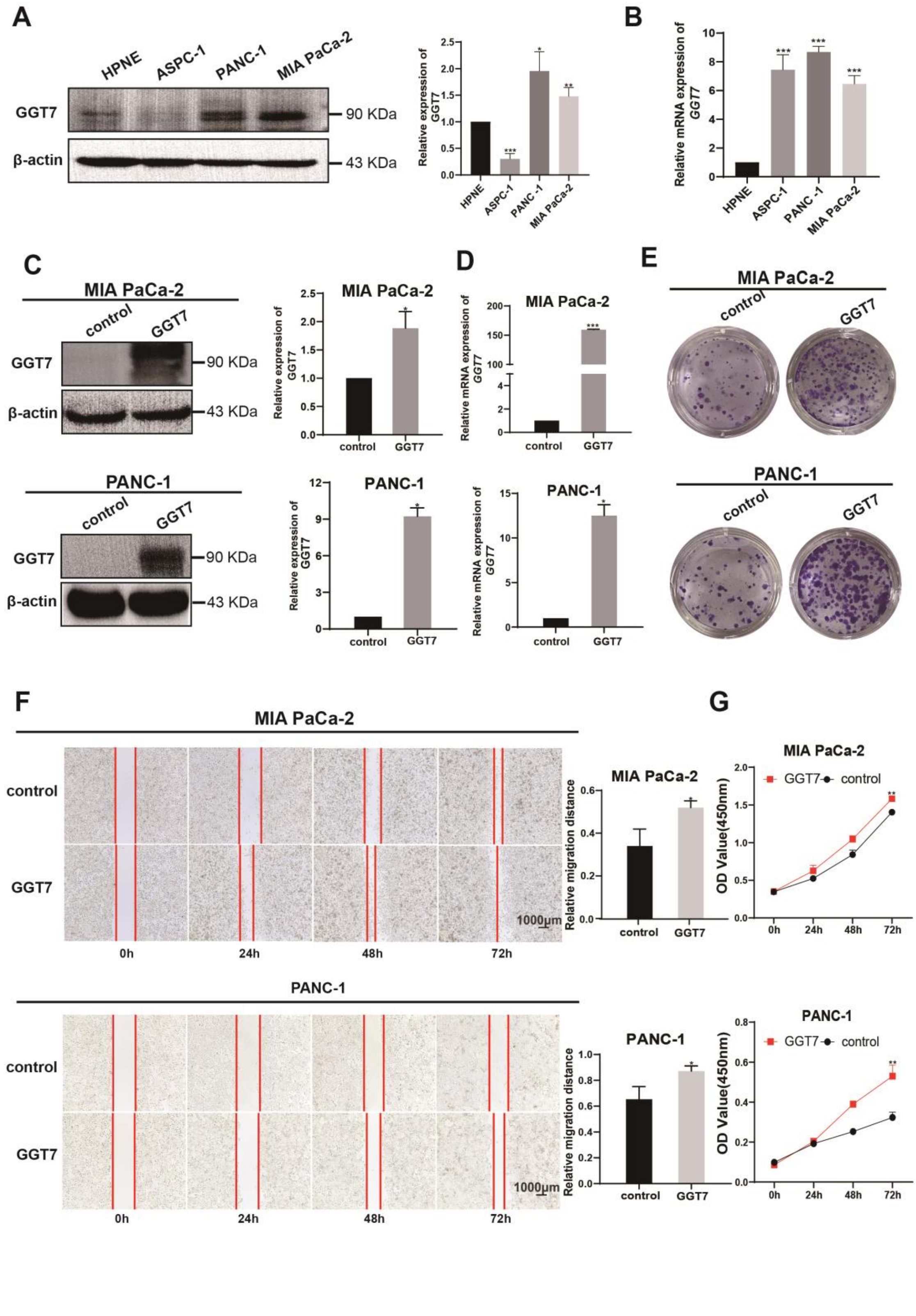
A
HPNE
ASPC-1
PANC-1
MIA PaCa-2
GGT7
90 KDa
β-actin
43 KDa
Relative expression of GGT7
B
Relative mRNA expression of GGT7
C
MIA PaCa-2
control
GGT7
PANC-1
D
E
F
1000μm
0h
24h
48h
72h
Relative migration distance
G
OD Value(450nm)

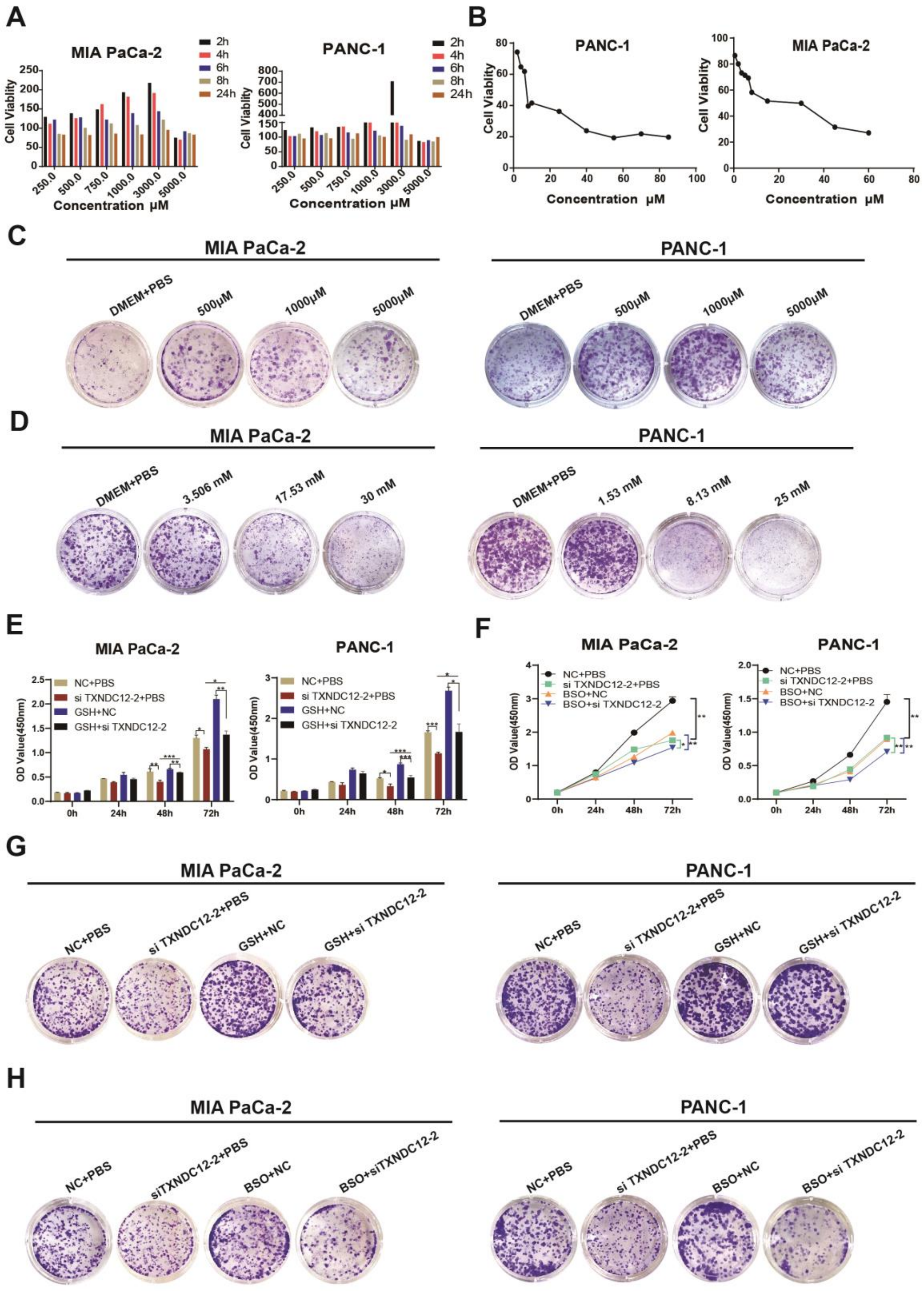
A
MIA PaCa-2
PANC-1
2h
4h
6h
8h
24h
Cell Viability
Concentration μM
250.0
500.0
750.0
1000.0
3000.0
5000.0
B
PANC-1
MIA PaCa-2
Cell Viability
Concentration μM
C
MIA PaCa-2
PANC-1
DMEM+PBS
500μM
1000μM
5000μM
D
MIA PaCa-2
PANC-1
DMEM+PBS
3.506 mM
17.53 mM
30 mM
1.53 mM
8.13 mM
25 mM
E
MIA PaCa-2
PANC-1
NC+PBS
si TXNDC12-2+PBS
GSH+NC
GSH+si TXNDC12-2
OD Value(450nm)
0h
24h
48h
72h
F
MIA PaCa-2
PANC-1
NC+PBS
si TXNDC12-2+PBS
BSO+NC
BSO+si TXNDC12-2
OD Value(450nm)
G
MIA PaCa-2
PANC-1
NC+PBS
si TXNDC12-2+PBS
GSH+NC
GSH+si TXNDC12-2
H
MIA PaCa-2
PANC-1
NC+PBS
siTXNDC12-2+PBS
BSO+NC
BSO+siTXNDC12-2
si TXNDC12-2+PBS
BSO+si TXNDC12-2
